# Identification of Motor Symptoms Related to Parkinson Disease Using Motion-Tracking Sensors at Home (KÄVELI): Protocol for an Observational Case-Control Study

**DOI:** 10.2196/12808

**Published:** 2019-03-27

**Authors:** Milla Jauhiainen, Juha Puustinen, Saeed Mehrang, Jari Ruokolainen, Anu Holm, Antti Vehkaoja, Hannu Nieminen

**Affiliations:** 1 Faculty of Medicine and Health Technology Tampere University Tampere Finland; 2 Unit of Neurology Satakunta Central Hospital Satakunta Hospital District Pori Finland; 3 Clinical Pharmacy Group Division of Pharmacology and Pharmacotherapy University of Helsinki Helsinki Finland; 4 Hospital Services Social Security Center of Pori Pori Finland; 5 Faculty of Management and Business Tampere University Tampere Finland; 6 Faculty of Medicine University of Turku Turku Finland; 7 Department of Clinical Neurophysiology Satakunta Central Hospital Satakunta Hospital District Pori Finland; 8 Faculty of Health and Welfare Satakunta University of Applied Sciences Pori Finland

**Keywords:** Parkinson disease, movement analysis, gait, wearable sensors, smartphone, home monitoring, mobile phone

## Abstract

**Background:**

Clinical characterization of motion in patients with Parkinson disease (PD) is challenging: symptom progression, suitability of medication, and level of independence in the home environment can vary across time and patients. Appointments at the neurological outpatient clinic provide a limited understanding of the overall situation. In order to follow up these variations, longer-term measurements performed outside of the clinic setting could help optimize and personalize therapies. Several wearable sensors have been used to estimate the severity of symptoms in PD; however, longitudinal recordings, even for a short duration of a few days, are rare. Home recordings have the potential benefit of providing a more thorough and objective follow-up of the disease while providing more information about the possible need to change medications or consider invasive treatments.

**Objective:**

The primary objective of this study is to collect a dataset for developing methods to detect PD-related symptoms that are visible in walking patterns at home. The movement data are collected continuously and remotely at home during the normal lives of patients with PD as well as controls. The secondary objective is to use the dataset to study whether the registered medication intakes can be identified from the collected movement data by looking for and analyzing short-term changes in walking patterns.

**Methods:**

This paper described the protocol for an observational case-control study that measures activity using three different devices: (1) a smartphone with a built-in accelerometer, gyroscope, and phone orientation sensor, (2) a Movesense smart sensor to measure movement data from the wrist, and (3) a Forciot smart insole to measure the forces applied on the feet. The measurements are first collected during the appointment at the clinic conducted by a trained clinical physiotherapist. Subsequently, the subjects wear the smartphone at home for 3 consecutive days. Wrist and insole sensors are not used in the home recordings.

**Results:**

Data collection began in March 2018. Subject recruitment and data collection will continue in spring 2019. The intended sample size was 150 subjects. In 2018, we collected a sample of 103 subjects, 66 of whom were diagnosed with PD.

**Conclusions:**

This study aims to produce an extensive movement-sensor dataset recorded from patients with PD in various phases of the disease as well as from a group of control subjects for effective and impactful comparison studies. The study also aims to develop data analysis methods to monitor PD symptoms and the effects of medication intake during normal life and outside of the clinic setting. Further applications of these methods may include using them as tools for health care professionals to monitor PD remotely and applying them to other movement disorders.

**Trial Registration:**

ClinicalTrials.gov NCT03366558; https://clinicaltrials.gov/ct2/show/NCT03366558

**International Registered Report Identifier (IRRID):**

DERR1-10.2196/12808

## Introduction

Parkinson disease (PD) is a progressive and degenerative disorder of the central nervous system, affecting both the physical and psychological health of the patient [1]. James Parkinson described the physical symptoms of the disease in 1817 as “the shaking palsy” [2], naming rest tremor as one of the distinguishing features. Rest tremor, rigidity, and bradykinesia (ie, the slowness of movement) are considered the main motor indicators of PD [1]. In addition to these motor symptoms, PD may affect the cognitive ability by causing dementia and indirectly affecting the mental health by increasing the risk of depression [1]. In recent years, nonmotor manifestations of PD have been given more recognition, and updates in the diagnostic criteria have been made to systematize the diagnostic process [3].

Motor symptoms are caused by the accumulation of alpha-synuclein protein in the central nervous system and the loss of dopaminergic neurons, thus reducing limb movement and control of the body. The symptoms usually start asymmetrically as a rest tremor or rigidity in one of the upper or lower limbs. The symptoms degrade the physical condition and decrease movement, thus affecting the quality of life. [1] In clinical practice, the symptoms are evaluated on a visual basis, but several studies have used wearable sensors and smartphones to identify the symptoms such as tremor [4-6], freezing of gait [7-8], and dyskinesia [9-10]. Although the symptoms can be identified visually, wearable sensors provide the possibility to monitor the patient remotely and collect more quantifiable data of the progression of symptoms. To our knowledge, there are two medical devices for monitoring of PD: The Personal KinetiGraph system for measuring bradykinesia, dyskinesia, tremor, and sleep [11-12] and the Kinesia Technology [13] for measuring tremor, dyskinesia, and mobility.

The diagnosis of PD is based on an interview, a physical examination, and an evaluation of the medical history of the patient by a physician. The consulting neurologist confirms the neuropathological indicators of the disease and rules out other conditions with similar manifestations using imaging and laboratory tests. If clinically needed, the symptoms are suppressed by prescribing dopaminergic drugs after the PD diagnosis is confirmed. When the disease progresses, a variety of medications and different therapy techniques must be used to help with the symptoms. [14] Patients visit their neurologists to adjust their medications, while control visits are organized constantly throughout the progression of the disease.

There are three symptomatic states in PD that depend on the level of medication. (1) The “Off” state implies that the dopamine level is too low in the central nervous system, thus increasing the cardinal features of PD: tremor, slowness, and rigidity. (2) The “On” state is the optimal dopamine level in the central nervous system, which implies very little or no sign of the disease, depending on how much the disease has progressed. (3) The overmedicated state is a hyperkinetic state in which the dopamine level is too high. This causes dyskinetic movements, fast walking and movements, and dystonic changes in the posture or limbs. [15] The effect of the medication is fast, but it may also wear off fast, before taking the next dose.

There are several challenges associated with diagnosing and monitoring PD with the current clinical practice. Clinical heterogeneity complicates the diagnosis because the patient may have a variety of symptoms of varying intensity and timing [14]. The symptoms present during the clinic appointment may not reveal all of the issues that are present at home, and the ability to cope independently may vary substantially between the On and Off states. On the other hand, prescribing the right dose of medication and scheduling doses to prevent Off states is challenging. Sudden fluctuation of On/Off states may be recognizable with wearable sensors that have automated algorithms [16]. To handle this challenge of recognizing fluctuations, remote monitoring at home would help health care professionals prescribe medication, and with complicated PD, it would help decide the optimal timeframe for secondary, invasive PD treatments such as deep brain stimulators or intraduodenal levodopa infusions.

In addition to the previous studies on identifying motor symptoms and current medical devices, we aim to build an affordable remote monitoring system using patients’ own devices, allowing long-term monitoring with minimal effort to the patient. The system is developed based on the findings from the data-collection process and future studies. This study is an observational case-control study that aims to collect a dataset from patients with PD and healthy controls by measuring their movements during daily life, using a smartphone and wearable sensors. The primary objective of the data analysis is to determine if PD-related motor symptoms can be detected and classified based on inertial signals recorded at home. Motor symptoms could also be used to determine whether the subject is in an early or progressed stage of the disease. The secondary objective is to distinguish changes in motor symptoms after a dose of the medication is taken. These changes should be visible with patients having distinct “On” and “Off” states in their motor symptoms. Home monitoring of patients with PD and automated data analysis could help health care professionals validate the level of motor symptoms and monitor the effects of medication. The long-term goal is to develop an affordable and easy-to-use solution for remote home monitoring of PD.

## Methods

### Inclusion and Exclusion Criteria

The inclusion criterion for the study group is the diagnosis of PD according to the International Classification of Diseases-10 code G20 [17]. All subjects were recruited from the Satakunta region in Finland. The common inclusion criteria for both the study and control groups were minimum age of 30 years, ability to walk at least 20 steps unassisted (use of assistive devices such as a walking stick were allowed), and no deep brain stimulation therapy during the study. In addition, the subject must not have been diagnosed with diseases causing symptoms similar to PD, such as multiple system atrophy, progressive supranuclear palsy, corticobasal degeneration, Lewy body dementia, or parkinsonism induced by a dopamine antagonist drug (ie, an antipsychotic drug such as metoclopramide). In addition, all subjects were required to speak and write fluent Finnish.

The control subjects are not specifically age matched to the patients, since both groups are recruited and studied at the same time and we aim to maximize the number of participants in both groups. However, recruiting control subjects from the family members of patients, for example, partners, balances the age distribution. Medical background information is collected from both groups to record conditions that affect the walking skills.

### Study Design

**Figure 1:**
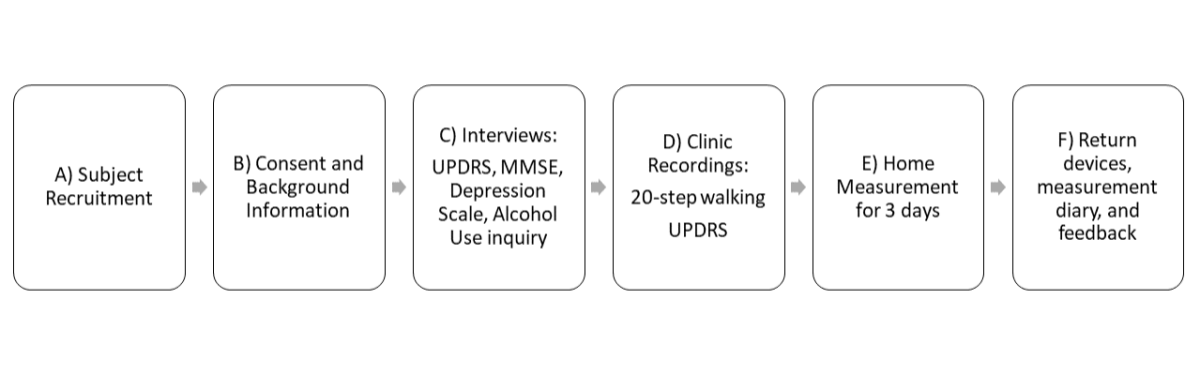
The study protocol consists of six phases, all of which are completed by both patients and control subjects. MMSE: Mini-Mental Status Examination; UPDRS: Unified Parkinson's Disease Rating Scale.

A study sample of 150 subjects will be recruited, of which 100 will be patients with PD and 50 will be healthy controls. Approximately 50 of the patients with PD will be in an early stage of the disease and the other 50 will be in a progressed stage of the disease. This classification is based on the modified Hoehn and Yahr Staging of PD [18], where patients with a score of 0-2 are considered to be in the early or mild stage of the disease, and patients with scores of 2.5-5 are considered to be in the progressed or more severe stage of the disease. Both the study and control groups go through the same measurement protocol, including a clinic visit with questionnaires and tests and a home measurement for 3 consecutive days. The home measurement period aims to record data showing short-term changes in walking patterns related to the On/Off state changes or wearing-off effect of medication. In addition, the period can be used to evaluate the subjects’ physical activity at home. Figure 1 presents the study protocol.

When a subject has been recruited, the study protocol begins with an appointment with a physiotherapist at Satakunta Central Hospital in Pori, Finland, or another appropriate location in the Satakunta region. First, the physiotherapist interviews the subject and collects his/her background information, diagnoses, medical histories, and current medications. Thereafter, the subject and physiotherapist fill in the structured questionnaires together. All the questionnaires were selected from official Finnish translations. The validated Finnish versions of the tests used in this study include the Alcohol Use Inquiry, C version [19]; the Depression Scale [20] to determine depressive symptoms; the Mini-Mental Status Examination [21] to determine the level of global cognition; and the Unified Parkinson’s Disease Rating Scale (UPDRS) [22] to determine the severity of PD symptoms. The UPDRS examination includes both interviews and physical examinations performed by the physiotherapist. The subject also performs a 20-step walking test while wearing all the sensors in addition to the physical examination performed during the UPDRS test [23]. During this 20-step walking test, the subject walks straight at his/her own pace for 20 steps in a hallway. The physiotherapist counts the steps silently and asks the subject to stop walking after 20 steps. The subjects do not count their steps, since rhythmic aids may help patients with PD to walk more normally. The timing of the previous PD medication is recorded for reference.

After the appointment at the clinic, the subjects wear the sensors for 3 consecutive days during their daily lives. The sensors are not worn during sleep or while showering or swimming. During these 3 days, the subject registers the intake time of all PD medications in a smartphone app as well as in a paper diary format. This manual recording was added, since the patients were having problems using the medication registration button despite the simple user interface. The double registration process ensures that medication intake is recorded as accurately as possible. The subjects also record other events, such as falls and other adverse effects, and feedback in the diary.

### Subject Recruitment

This study recruits subjects through two routes. In the first route, patients are recruited from Satakunta Central Hospital. These subjects are outpatients who visited the hospital due to PD in the past 5 years. The study physiotherapist sends an informational letter to the subjects and follows up by phone 1 week later. The physiotherapist introduces the study and asks if the patient is willing to participate. If the patient is willing to participate, the physiotherapist sets up an appointment at Satakunta Central Hospital.

In the second route, the physiotherapist and the responsible investigator (neurologist) visit local PD communities in the Satakunta region and give lectures on the study and its intentions. After the presentation, the patients and their family members (partners) have the opportunity to sign up for the study in either the study or control groups. If many volunteers sign up, the physiotherapist may organize appointments at clinics in these locations to minimize travel costs and effort from the patients.

If the study cohort is incomplete after these recruitment processes, an advertisement is published in the local newspaper, as well as the Satakunta Central Hospital newsfeed, to inform potential subjects about the study and ask for participation. Hospital staff and the research consortium were recruited as the control group, in addition to the family members of the study subjects.

Since the study is solely based on recruiting volunteers, there is a risk of some bias in the study cohort, both amongst patients and controls. It is difficult to recruit subjects with a lot of health problems or with very severe PD symptoms. For example, subjects with depression or severe cognitive issues may not want to participate, even if their physical condition is appropriate for the study. However, our aim is to include all the volunteers fulfilling the inclusion criteria into the study and consider the properties causing bias in the analysis phase.

### Roles of the Research Staff in Data Collection

The physiotherapist involved in this study is a trained clinical physiotherapist who works with neurological patients in the hospital. The physiotherapist is responsible for contacting the subjects, scheduling appointments, conducting measurements, instructing the subjects to use the devices, and cleaning the measurement devices after each use. The subjects may contact the physiotherapist as well as the researchers to provide more information and ask for help when using the devices. All the devices are provided by the research group. Therefore, the subjects do not need their own smartphones or other devices in the study. The study smartphone is only used for data collection and not for any personal use.

A researcher at Tampere University in Tampere, Finland, assists in conducting the measurement protocol at the hospital and monitors the operation of the data-collection system. The data-collection system consists of sensors that measure the data and a smartphone that buffers and sends data through an internet connection to a server located at Tampere University, where the researcher inspects the data on a personal computer. The researcher assists the physiotherapist with the devices, if any problems occur.

### Ethical Approval and Trial Registration

This study is conducted according to the Ethical Principles for Medical Research Involving Human Subjects, as stated in the 2013 revised version of the 1964 World Medical Association Declaration of Helsinki. All information collected by Tampere University is coded and does not contain identifying information. The identifying data are held appropriately in a locked storage facility at Satakunta Hospital District, and only the study physiotherapist and the responsible investigator are allowed to view them.

A favorable statement was obtained for the study protocol from the Ethics Committee of the Hospital District of Southwest Finland in Turku [24] on October 24, 2017 (ETMK 101/18012017). Minor changes to the study protocol were made based on tests of the measurement protocol with 10 pilot subjects. These changes were approved by the ethics committee in April 2018. For example, the UPDRS test was extended to the healthy control group. The changes to the measurement protocol are further discussed in the Results section of this paper.

In addition to the ethical committee statement, the National Supervisory Authority for Welfare and Health (Valvira) in Helsinki, Finland, approved the study in April 2018 (identification number 394-2018).

The KÄVELI study is registered at ClinicalTrials.gov (NCT03366558) [25].

### Sensors Used in the Study

Data are collected using three types of sensors: built-in inertial sensors in a mobile phone (Nokia 6, HMD Global Ltd, Finland) provided by the study, a wrist-worn wearable device measuring a gyroscope signal (Suunto Movesense, Suunto Ltd, Finland [26]), and a smart insole measuring ground reaction forces in the feet (Forciot smart insole, Forciot Ltd, Finland [27]). The primary measurement device is the smartphone, since it is used both in the clinic and at home for all subjects. The other two devices are used mainly in the clinic, but the Forciot insole is also tested in home settings with a few subjects who were comfortable using the additional device.

**Figure 2:**
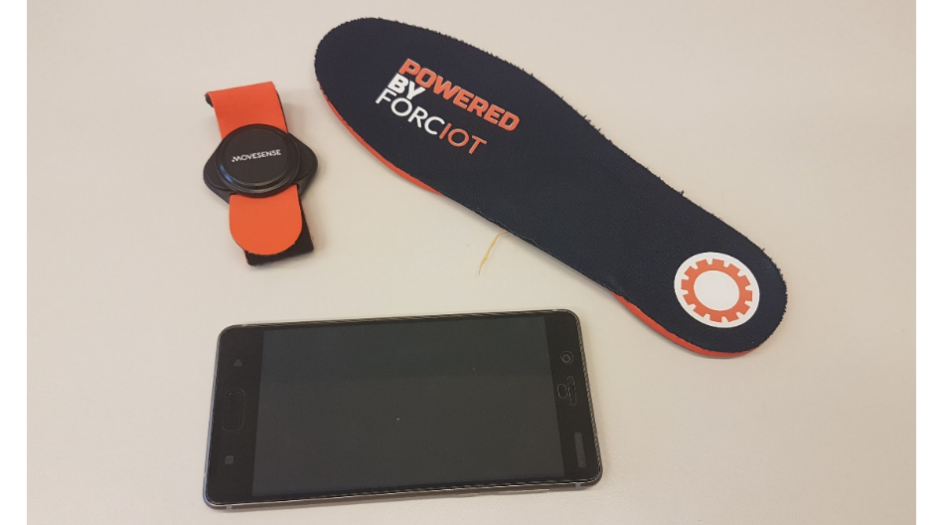
Sensors used in the study: Movesense smart sensor (upper left corner), Forciot smart insole (upper right corner), and Nokia 6 smartphone (lower left corner).

The subjects also registered their PD medication intake in a smartphone app set to the home screen of the phone. However, to ensure that the medications are correctly registered, the subjects are asked to write their PD medication intake in a manual diary format as well. Figure 2 presents the sensors used in this study.

The smartphone contains built-in sensors for acceleration, angular velocity, and orientation, which are used to measure the movements of the phone while it is carried by the subject, thus providing information about their movement dynamics.

While the subject is wearing the device on the waist, the acceleration describes the changes in velocity and orientation of the subject in the linear direction, and cyclic movements such as walking are visible as cyclic changes in the acceleration. The gyroscope describes the rate of changes in orientation in relation to the center of the phone. The orientation signal describes the orientation of the phone in three dimensions, in which the phone orientation can be checked. This signal helps to check if the subject accidentally wears the phone in a different position than instructed.

**Figure 3:**
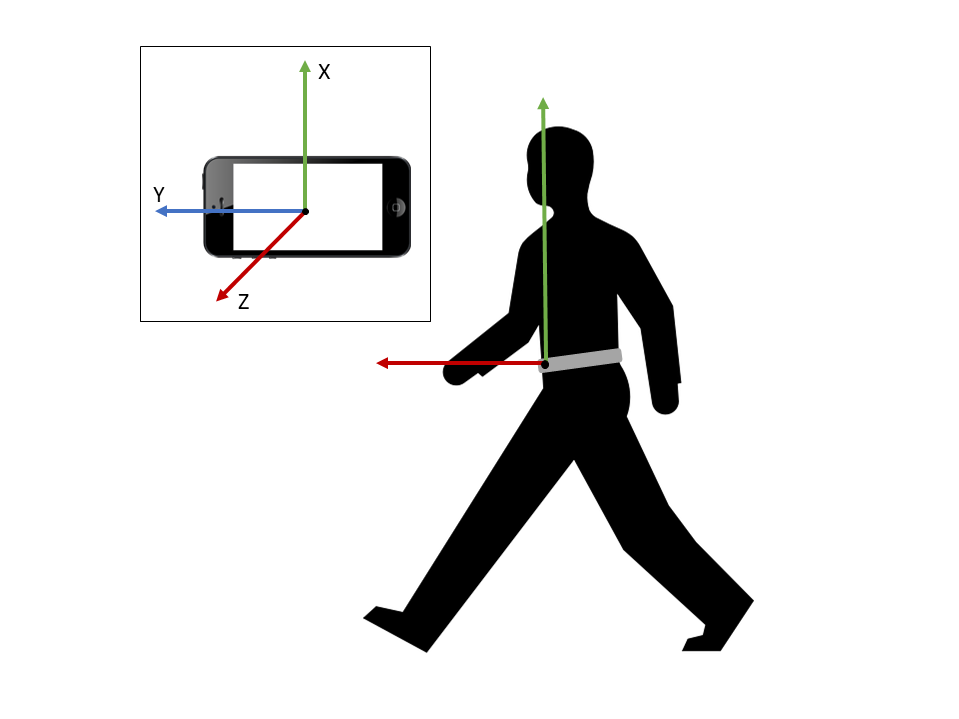
The orientation of the smartphone when worn on the waist. The X-axis measures vertical movement, the Y-axis measures horizontal movement in the lateral direction, and the Z-axis measures movement in the posterior-anterior direction. The phone is worn in a bag on the waist and inserted correctly on the stomach side with the screen out and the right side up.

These sensors are available in most smartphones and are used, for example, in mobile games, compasses, and step counters. The smartphone is located in a waist-worn small bag in front of the stomach. Use of the bag ensures that the device can be worn regardless of the type of clothing; thus, no pockets are needed. The placement was set in front, so that it would not disturb the subject when sitting or lying down. The phone is oriented horizontally with the positive X-axis facing upward and the positive Z-axis facing forward. Figure 3 presents the phone’s orientation and location.

The data recorded by the sensors are further used to classify the subject’s walking patterns. We selected the Android 8–based Nokia 6 smartphone for data collection because of its ability to effectively disable all power-management settings. This is an important feature to successfully collect long-term data. The instantaneous sampling frequency of the sensors varies between 50 Hz and 100 Hz due to implementation of the data-collection framework. All the signals measured with the smartphone are resampled to 100 Hz during signal processing. The measurement ranges for the accelerometer and the gyroscope are ±16 g and ±34.9 radians/second, respectively, and their resolutions are 14 bit and 16 bit, respectively; in addition, their internal peak-to-peak noise level ranges were found to be roughly ±1 mg and ±0.005 radians/second, respectively.

Suunto Movesense, manufactured by Suunto Oy [26], is a heart rate– and movement-monitoring sensor worn on the wrist in this study during the physical examination of the UPDRS. The device communicates with the mobile phone using Bluetooth low energy, and the sampling frequency of Movesense is 52 Hz. The measurement range is ±34.9 radians/second. The data provided by this sensor can be used, for example, to measure the rest tremor and detect attenuated arm swing during walking, which is a typical symptom in PD.

The Forciot smart insole, manufactured by Forciot Oy [27], is a pressure-sensing insole prototype that includes 23 sensing elements distributed, so that they cover the whole area of the sole with a sampling frequency of 50 Hz. The smart insole also calculates the applied total force by integrating the data of the individual sensing elements. One insole is used during the UPDRS examination at the clinic appointments. Two subjects also used a pair of insoles during the home measurements. Thus, the insole provides information on the walking pattern and pressure distribution in the feet.

### Data Collection and Management

**Figure 4:**
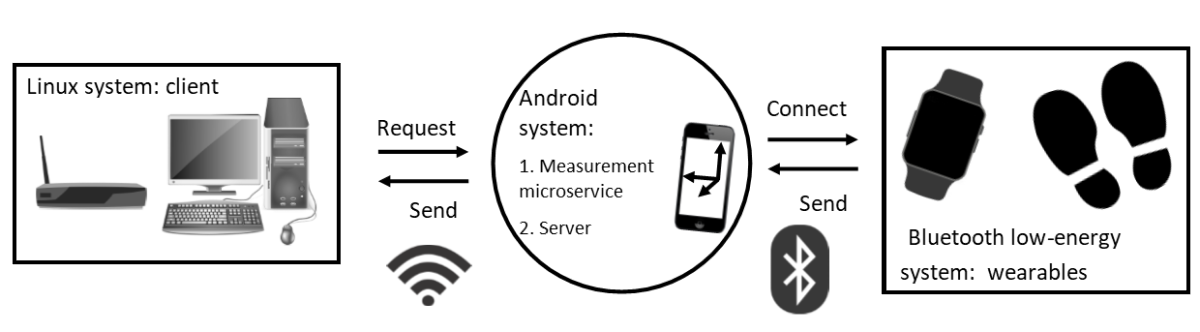
An overview of the structure of the data-collection system. The system consists of three independent systems: (1) a Linux system, (2) an android system acting as a temporary server, and (3) a Bluetooth low-energy system consisting of independent wearable devices.

This study introduces a novel perspective on the mobile microservice architecture in a data-transfer system [28]. In this system, all sensors and wearable devices act as independent services with their own architecture. However, with a common interface between the server and the services, data can be collected simultaneously and asynchronously. In fact, the entire front-end, back-end concept is flipped because the smartphone acts as a server behind the simple user interface, and the personal computer acts as a client who is requesting information according to the data-collection frequency set by the personal computer user. Figure 4 presents an overview of the data-collection system.

The smartphone worn by the subject collects information from all the sensors and stores it temporarily. The size of the buffer on the phone is adequate for storing data of up to 1 hour, but after that time, the system will start to overwrite the data. The wearable devices are paired with the smartphone in the beginning of a recording, but after a momentary loss of connection, they will reconnect automatically. The personal computer for data collection acts as a client and requests information from the smartphone via an internet connection by using the smartphone’s internet protocol address. In this study, the frequency for requesting the signals is 5 minutes. The client receives the information via the internet connection and stores the data in text files. Several subjects can be measured simultaneously because each subject only requires one smartphone and the wearable devices. The personal computer for data collection can also save several data-collection streams simultaneously within the Linux operating system.

There are two approaches to securing information during data transfer: (1) encrypting the information before sending it through an unsecured connection and (2) building a secure connection between a smartphone and a router that cannot be accessed by any internet protocol addresses other than the ones used by the data-collection smartphones and the client’s personal computer. The former approach was tested at the beginning of the study, and the data were collected successfully in an encrypted form. However, a secure connection improves efficiency during data management and signal processing, because the files are ready to use immediately after data collection. The second solution was also implemented and tested during the project in cooperation with a local telecommunications company. However, no identifying information is transmitted through the internet connection. The subject identification and smartphone are accurately linked manually before analyzing the data. This implementation will be more thoroughly discussed in future publications related to the data-collection system.

This database will be granulized and compressed into a format that can be used in further analyses, including structuring the information, developing the metadata, and ensuring anonymity of the data. For example, rather than knowing the exact date of the measurement, only the time of the day and season will be recorded when analyzing walking habits.

### Data Verification

Data verification requires confirming that the measured signals have the appropriate quality to accurately distinguish walking segments from other movements and to use the signals for further analysis. The possible issues faced during home or clinic measurements are also recorded and analyzed, including, for example, usability issues with the technology as reported by the participants or network issues leading to the loss of data. These analyses would confirm that there is an appropriate amount of high-quality data that is usable in machine learning applications. If any such problems are identified in the measurement setup, they will be fixed to ensure the quality of the data.

The collected signals are checked both visually and statistically to assess whether they contain the necessary information for the classification of different types of movements. A minimum sampling frequency of 50 Hz was required for the measurements conducted with the smartphone based on the previous studies presenting human walking under 10 Hz [29] and hand tremors in PD under 20 Hz [30]. The measurements will be discarded if they are below a 50-Hz sampling frequency. Suunto Movesense and Forciot use their own sampling frequencies of 52 Hz and 50 Hz, respectively.

Information is collected on how many files were successfully recorded during a 3-day measurement. Successful measurements are defined as those that are recorded and transferred correctly and have the appropriate sampling frequency. This information is used to monitor how well the measurement system functions when the subject is responsible for merely charging the smartphone and restarting it if the power runs out. The performance analysis includes battery life, possible network issues, and usability issues. These properties are studied and used in further development of the measurement system.

### Data Analysis

Methods for data analysis are developed and structured based on a data-analysis pipeline. This pipeline includes data preprocessing, walking detection, calculation of features related to changes in walking, and feeding the features into a machine-learning system to classify the symptoms and subjects. The classification results and the performance of the classifier are then analyzed. The main features of data analysis include the identification of patients with PD from healthy subjects based on sensor data and prediction of UPDRS scores from the sensor data. The long-term target is to build an automated classification system that could support home monitoring by using machine learning methods. All the data analysis will be done in Python 3 (Python Software Foundation, Wilmington, DE) and MATLAB (The MathWorks, Inc, Natick, MA).

We will be using a data-driven approach to analyze and compare a large number of features calculated from the signals of the different sensors. However, we will consider possibilities to combine the information of different sensors to define such features or parameters that are likely relevant to PD, for example, the amount of arm swing during walking. The incompleteness of the data set (wrist and insole sensors not included in all the recordings), however, limits the use of parameters requiring combining sensor information. This will be discussed in future publications on the data analysis.

The preprocessing phase includes the signal-processing steps that are required before the signals can be used in further data analysis. The signals are resampled to obtain a constant sampling frequency; simultaneously measured signals are aligned to have the same starting and ending time; and, if necessary, the signals can be filtered with a basic low- or high-pass filters.

An accurate walking-detection algorithm and careful selection of machine-learning features are important when developing the data-analysis pipeline. Activity-detection algorithms have already been developed by other research groups, for example, based on the mean amplitude deviation [31-32], which is more thoroughly defined in another previous study [33]. An algorithm based on the existing literature will be developed and implemented during this study. The walking-detection algorithm may extract either walking segments or individual steps. When recognizing the walking patterns from home data, we may use the 20-step walking tests as a personal reference of the subject’s walking style. Walking detection from the 20-step tests is rather easy, since the signals only contain walking or standing. Walking detection will be thoroughly analyzed and discussed in future publications considering the analysis of data from home measurements.

The selection of features is also important when building a machine-learning system for health care use. The features should describe real physiological changes in the walking patterns; for example, if the patient is in the On state with good balance in the medication, his/her walking is classified as better than the same patient without the medication (Off state). Although On and Off states are not specifically defined in this study, we aim to recognize the differences before and after taking the medication. Features can be selected with a statistical approach, for example, by applying the minimum Redundancy Maximum Relevance algorithm [34] or the theoretical understanding of PD to choose features related to the physiological changes in the patients. Features may include both statistical features of the signals such as mean, median, or SD, or they can be defined from the physiology and be based on traditional features used in gait analysis, such as step length or speed.

The subjective UPDRS classification performed by the physiotherapist is used to test the classification of the subjects from the 20-step walking tests. The UPDRS score can be used as the true label of the subject’s current state, and the walking tests are classified into several groups identifying the severity of motor symptoms in walking. In home measurements, the UPDRS score defined at the clinic may be used to test whether the classification result before taking the PD medication differs from that soon after taking the medication. Alternatively, unsupervised learning methods such as clustering approaches may be used to study whether there are differences in the feature values before and after taking the medication.

The classification phase includes applying different machine learning classifiers to study the most feasible method for classifying the symptoms and changes in symptoms related to medication intakes. For example, a support vector machine [35] and random forest [36] will be used to classify walking patterns. Classification performance is evaluated by a confusion matrix and the metrics related to it including accuracy, sensitivity, specificity, and the receiver operating characteristic curve. Other measures may be added to evaluate the performance more thoroughly.

The data analysis aims to develop methods to answer the following hypotheses: (1) Can we distinguish patients with PD from healthy controls by using wearable sensors and machine learning classifiers? (2) Can we assess the severity of symptoms before and after medication intake? These key hypotheses may be further specified or expanded in further analyses. These methods, combined with the novel data-collection method, can be further developed into a home-monitoring system for health care professionals and neurological patients.

## Results

Data collection began in March 2018 by testing the measurement protocol with 10 subjects. These subjects will be included in the final study sample. Minor improvements were made to the measurement protocol based on the first subject’s recordings in the clinic and in-home measurements. The changes included (1) the double registration of medications, as discussed in the Methods section of this paper, and (2) the decision to conduct the UPDRS questionnaires and tests with the control subjects, even though they were not expected to have PD symptoms.

All three measurement devices were planned to be used in the home measurements, but due to the technical requirements of Suunto Movesense [26] and Forciot [27] insole, they were not implemented in the protocol for data collection at home. Forciot [27] insoles were tested with subjects for 1 day prior to the 3-day home measurement. Two subjects were tested during the study. This is because the insoles are operated by a battery, which only has a lifetime of 24 hours. In addition, Suunto Movesense [26] was connected using a Bluetooth connection, and the connection was not sufficiently stable to be used at home. Therefore, the device was only used in the clinic measurements.

After the pilot study, the capacity of simultaneous studies increased from 3 to 10. Most of the subjects were recruited from the PD community visits where there was a lot of interest to participate in the study. A secure internet connection was set up for data transfer by building a private mobile network for the research project in cooperation with a local telecommunications company. This network was tested and implemented in August 2018. After the update, encryption of the measurement data was not needed because the network was only accessible by devices in the study. By the end of December 2018, we collected data from a total of 103 subjects, 66 of whom are patients with PD and 37 are control subjects. Subject recruitment and data collection will be continued in the spring of 2019.

## Discussion

This paper describes the research protocol for a PD-monitoring study involving the use of smartphones and wearable sensors, also known as the KÄVELI project. This study aims to gather a dataset of 150 subjects to develop methods for monitoring PD-related motor symptoms in daily life. The user-friendly measurement setup is enabled by a data-collection system that consists of several microservices that automatically collect movement information when a subject wears the measurement device and registers their PD medication intake manually and through a smartphone app. There has been an increasing trend in studies toward home monitoring of PD with, for example, smartphones [7,23,37-39] and other wearable sensors [4,8,37,40-42]. In addition, several research groups have developed algorithms for recognizing various types of movements such as walking, running, and climbing stairs [31,32,43-45]. Based on the literature, there is a potential for monitoring PD with wearable solutions and machine-learning principles. These principles are applied in order to recognize the descriptive features of walking segments as well as to determine what sensors are required to achieve accurate recognition of the symptoms and evaluate their severity. The selection of correct features is important for linking the results to real physiological changes that are evident in the subjects’ walking.

Some mobile health platforms have been proposed to monitor PD patients during preset tests, such as PD Dr [6] and PD_manager [46], both of which have been tested on a relatively small set of patients with PD. HopkinsHD [47] is collecting data from free-living people and has been tested on a set of 226 subjects, both control subjects and patients with PD. These platforms have resulted in indications that home monitoring of PD patients is feasible. Our aim is to provide a platform that is suitable for long-term monitoring and uses the patients’ own smartphones or very affordable wearable sensors.

Most of the previous studies concentrating on the recognition of PD symptoms with wearable sensors have been conducted with relatively small datasets (10-50 subjects in total), which are feasible for proposing new methods in data collection or analysis, but not enough to validate the system with a larger variety of subjects. Some studies, however, have recruited more subjects [23,37,47]; these studies have collected larger datasets of several hundred subjects. Larger studies will be needed to validate systems before implementing them into a commercial product. The dataset collected in the KÄVELI project will be valuable in the investigation of PD motor symptoms in a quantitative manner, whereas the present clinical monitoring of PD is based on the subjective information provided by the patient and clinical observations by a neurologist or physiotherapist [25]. Despite the opportunities and new innovations presented by wearable sensors and machine learning, home monitoring has several practical challenges that must be evaluated and solved before they can become a common part of clinical routines. First, independent use of technological devices may create difficulties among the elderly, and acceptance of technology takes time and special effort in usability. Second, even if the technology itself is not new to the patients, severe motor symptoms and Off states combined with cognitive and memory disorders may complicate the registration of medication intakes or other tasks given to the patient [48]. Finally, even though home monitoring allows for measurements to take place in real-life situations that provide a more extensive view of symptoms, ensuring adherence to the measurement protocol is impossible outside of the clinical environment. For example, subjects can be reminded to register their medication intake, but they may forget to register it anyway, or the placement of the device might be changed unexpectedly.

The KÄVELI project’s measurement protocol aims to minimize these challenges by developing a user-friendly interface for the smartphone that registers medication intake and by providing written and spoken instructions for the use of all devices included in the study. The level of instruction also aligns with the subject’s confidence with technology. This is assessed by a question in the background interview: “How confident are you in using a smartphone?” which is also recorded in the study material. In addition, the physiotherapist can be reached by phone for further instruction if any challenges occur during the measurement period.

Despite the simple user interface of the smartphone (only one visible button for the medication recording), some subjects have had issues using the smartphone. Therefore, medications were also recorded in a paper format, which led to extra work for the subjects. This limitation, as well as any adverse effects or usability issues, will be recorded and considered in the development of the measurement system.

One potential limitation to the study is the recruitment of control subjects. It may be difficult to collect a dataset with a variety of mobility skills and still match the age group of patients with PD. We have recruited control subjects from among the patients’ family members, but this might also be considered a limitation. These challenges must be addressed accordingly when carrying out the study.

In conclusion, this study will provide quantitative information on PD motor symptoms and their statistical properties. The collected dataset will be used to develop algorithms and create tools for remote monitoring of PD progression by physicians and to assist with adjusting the medication.

## Abbreviations

PD: Parkinson disease
UPDRS: Unified Parkinson's Disease Rating Scale
